# Facilitators of and barriers to County Behavioral Health System Transformation and Innovation: an interview study

**DOI:** 10.1186/s12913-024-11041-9

**Published:** 2024-05-09

**Authors:** Xin Zhao, Rachel Varisco, Judith Borghouts, Elizabeth V. Eikey, David Safani, Dana B. Mukamel, Stephen M. Schueller, Dara H. Sorkin

**Affiliations:** 1 Department of Psychiatry and Behavioral Sciences, University of Washington, Seattle, USA; 2 Seattle Children’s Research Institute, Seattle, USA; 3 Department of Medicine, University of California, Irvine, USA; 4 Herbert Wertheim School of Public Health and Human Longevity Science, University of California San Diego, La Jolla, USA; 5 The Design Lab, University of California San Diego, La Jolla, USA; 6 Department of Psychiatry and Human Behavior, University of California, Irvine, USA; 7 Department of Psychological Science, University of California, Irvine, USA

**Keywords:** System transformation, Behavioral health, Value-based payment, Payor-agnostic care, Digital resource navigation

## Abstract

**Background:**

Inadequate and inequitable access to quality behavioral health services and high costs within the mental health systems are long-standing problems. System-level (e.g., fee-for-service payment model, lack of a universal payor) and individual factors (e.g., lack of knowledge of existing resources) contribute to difficulties in accessing resources and services. Patients are underserved in County behavioral health systems in the United States. Orange County’s (California) Behavioral Health System Transformation project sought to improve access by addressing two parts of their system: developing a template for value-based contracts that promote payor-agnostic care (Part 1); developing a digital platform to support resource navigation (Part 2). Our aim was to evaluate facilitators of and barriers to each of these system changes.

**Methods:**

We collected interview data from County or health care agency leaders, contracted partners, and community stakeholders. Themes were informed by the Consolidated Framework for Implementation Research.

**Results:**

Five themes were identified related to behavioral health system transformation, including 1) aligning goals and values, 2) addressing fit, 3) fostering engagement and partnership, 4) being aware of implementation contexts, and 5) promoting communication. A lack of fit into incentive structures and changing state guidelines and priorities were barriers to contract development. Involving diverse communities to inform design and content facilitated the process of developing digital tools.

**Conclusions:**

The study highlights the multifaceted factors that help facilitate or hinder behavioral health system transformation, such as the need for addressing systematic and process behaviors, leveraging the knowledge of leadership and community stakeholders, fostering collaboration, and adapting to implementation contexts.

**Supplementary Information:**

The online version contains supplementary material available at 10.1186/s12913-024-11041-9.

In the United States, the system of providing and coordinating behavioral health services is inefficient. Health care systems are largely paid via a “fee-for-service” model that incentivizes increasing the number of billable hours rather than improving patient outcomes and quality of care [1]. At the system level, provider shortage, disparities in insurance coverage, and the existing fee-for-service reimbursement model contributed to longstanding unmet service needs [2–5]. At the individual level, behavioral health stigma, limited mental health literacy, and lack of knowledge about appropriate resources challenge people’s ability to navigate and access behavioral health resources, especially among historically marginalized and uninsured groups [3].

In light of the multilevel barriers hindering access to behavioral health services, system transformation is needed. According to California Health Interview Survey reports, one in five Orange County residents reported they needed, but did not receive, behavioral health support [6]. Those from disadvantaged socioeconomic backgrounds were less likely to access behavioral health support than those with more resources. The unmet need within the County behavioral health system can be improved if providers and patients have access to information about care (efficient resource navigation) and patients get linked with value-based behavioral health services regardless of their insurance status (payor-agnostic care). Orange County’s Behavioral Health System Transformation (BHST) Innovation Project in California aims to create a patient-centered system where all residents in Orange County can be served regardless of their insurance status and clinical needs. An innovation project introduces a new practice or approach in the field of behavioral health with a primary focus on learning or process change. The BHST Innovation Project includes two parts: developing a template for value-based payment contracts that promote payor-agnostic care (Part 1) and developing a public-facing digital platform (OC Navigator) to increase access to information and support resource navigation (Part 2) [6]. Innovation projects are limited to a maximum of five years, with the expectation that successful projects should transition to integration into standard practices and sustainment. The data for this paper come from the first half of a five-year innovation project. Thus, the current paper focuses on early lessons learned regarding facilitators and barriers.

## Backgrounds for components of the BHST Innovation Project

### Part 1. Developing a template for value-based payment contracts that promote payor-agnostic care

Value-based payment models tie payments for services to the quality of care and patients’ clinical outcomes rather than the volume of services delivered [7]. Since 2016, several states in the US, such as Washington, New York, Minnesota, Maine, and Massachusetts, made attempts to implement value-based care [1, 8–12]. In Washington and New York, transitioning to value-based care improved the quality of behavioral health services. In Washington State, a value-based care initiative targeted the implementation of the Collaborative Care Model, an evidence-based team approach to behavioral health interventions in primary care. In this program, 25% of funding to participating community clinics was contingent on meeting value-based payment targets (i.e., providing evidence-based care) and active participation in the program [8]. Compared to patients enrolling in the program without the value-based components, those enrolling in the program with value-based components were more likely to have depression outcomes that improved in a shorter amount of time (Bao et al., 2017). New York State Collaborative Care Medicaid also used a value-based program. In this program, 25% of the monthly, patient-level case-rate payment was withheld each month and was paid retroactively after six months for patients who had clinical improvement or had their treatment plan adjusted in response to a lack of clinical improvement [12, 13]. This program led to an increase in the proportion of patients screened for depression and patients who showed clinical improvement after 10 weeks of treatment at participating sites, compared to before launching the program. In addition to improving the quality of care, value-based payment models also have the potential to help address challenges faced by the traditional fee-for-service model, such as overutilization of services and high costs [7, 14].

Despite the reported positive implementation outcomes of the value-based payment initiatives, the process of implementing and sustaining value-based payment models is often challenging and varies largely by state [7, 10]. In the United States, buy-in of value-based payment models from commercial payors is challenging. For example, qualitative analyses of interview data revealed limited interest in adopting value-based contracts with providers among commercial payors in Arkansas, Maine, and Minnesota [10]. Specifically, commercial payors expressed concerns about (1) the need for tailoring a value-based contract to align with just one state when they have business in multiple states; and (2) market competition, such as subsidizing the care of patients covered by other payors who did not make similar investments to adopt value-based payment models [10].

Payor-agnostic care allows all patients to be served regardless of their insurance status and clinical needs, as it prioritizes patient needs and outcomes above financial profitability. A payor-agnostic model typically includes supporting uninsured individuals. Funding sources for uninsured individuals might include self-pay options, philanthropic donations, and government grants. The barriers to and facilitators of multi-payor alignment have been more studied in primary care settings. One notable effort is the Comprehensive Primary Care (CPC) initiative launched by Centers for Medicare & Medicaid Services (CMS), which is one of the largest multi-payor initiatives [15]. Interviews from CMS staff, CPC-participating payors, and stakeholder organizations described that competitive market dynamics and competing institutional priorities were barriers to multi-payor and multi-sector collaboration. Leveraging champion support and seeking input on decisions related to system transformation from key community stakeholders helped build trusting relationships and align different payors. In the sphere of behavioral health, emerging efforts of moving towards payor-agnostic care hold great potential in ensuring equitable health care access. However, barriers to and facilitators of implementing payor agnostic behavioral health care are less known. One example was the Blue Shield Health Reimagined pilot program. In this program, Blue Shield embedded Community Health Advocates in ten primary and specialty care practices to provide payor-agnostic care to support individuals who were not Blue Shield members to receive services [16]. In the first fourteen months of the program, a large and diverse population was served (i.e., *N* > 1,900 patients, > 30% Latinx/Hispanic) across four participating regions in California (Paulson et al., 2021). Paulson et al. (2021) analyzed focus group and interview data to identify facilitators of and barriers to embedding Community Health Advocates within primary and specialty care practices to provide payor-agnostic care, with a focus on intervention implementation. Overall, to improve access to behavioral health care access, additional work is needed to understand facilitators of and barriers to promoting payor-agnostic care.

### Part 2. Developing a digital platform to increase access to information and support resource navigation

A digital resource navigator is a public-facing digital platform that serves as a resource directory. A great digital resource navigator can improve the efficiency of resource navigation, care coordination, and knowledge sharing by speeding up communication among different sectors and reducing the need for human labor. Past work on digital resource navigation has mostly focused on supporting care coordination for patients and providers who are already situated in the care system, such as through the use of the electronic health record and web-based communications [17]. Much less work has focused on knowledge sharing and information exchange of service options before patients connect with a provider in the behavioral health sphere. One exception was the **M**ental health **I**ntelligent information **R**esource **A**ssistant (MIRA), a web-based conversational chatbot developed in Canada during the COVID-19 global pandemic [18]. MIRA was developed to provide individuals with (1) information on substance use and mental health and (2) information on behavioral health services in Canada. This digital resource navigation tool is publicly available and informed by subject experts. As described by their published protocol, data collection was anticipated to take place from May 2022 to May 2023 [18]. However, no published work is available regarding provider and stakeholder perceptions of such tools. A digital resource navigator presents a scalable opportunity to streamline information sharing and improve access to care, although further exploration of facilitators of and barriers to developing and implementing such tools is needed.

## Consolidated Framework for Implementation Research (CFIR) framework

CFIR is a comprehensive framework that can capture innovation-related factors and the complicated contextual factors that may influence implementation of an innovation. The initial version of CFIR comprised 39 subdomains grouped into the following five broad domains, including (1) innovation characteristics, (2) inner setting, (3) outer setting, (4) individual characteristics, and (5) process factors [19]. Innovation characteristics refer to characteristics of the template for value-based contracts (Part 1) and the digital resource navigator (Part 2), such as the perceived innovation source, complexity, evidence strength and quality, and relative advantage. Inner setting refers to the context in which the innovation takes place (in this study the Orange County Public Behavioral Healthcare System) including factors such as compatibility, leadership engagement, and networks and communication. Outer setting refers to the wider economic and social context that influences the innovation, such as federal and state policies and external incentives. Process refers to the steps taken during the innovation and implementation process, such as engaging and planning. Individual characteristics refer to the values and views of individual users of the innovation. Adapted definitions of CFIR constructs are presented in Table 1.

**Table 1:** Final Codebook Guided by Consolidated Framework for Implementation Research

Domain	Construct	Definition
Inner Setting	Innovation Climate	The absorptive capacity for change, shared receptivity of involved individuals to an innovation, and the extent to which use of this innovation will be rewarded, supported, and expected within Orange County
	1. Tension for Change	The degree to which stakeholders perceive the current situation as intolerable or needing change Include statements that (do not) demonstrate a strong need for the innovation and/or that the current situation is untenable
	2. Compatibility	The degree of tangible fit between meaning and values attached to an innovation by involved individuals, how those align with individuals’ own norms, values, and perceived risks and needs, and how the innovation fits with existing workflows and systems
	3. Relative Priority	Individuals’ shared perception of the importance of the implementation within the organization Include statements that reflect the relative priority of developing this innovation and related planning efforts
	Readiness for Implementation	Tangible and immediate indicators of organizational commitment to its decision to developing this innovation and related planning/implementation efforts
	1. Leadership Engagement	Commitment, involvement, and accountability of leaders and managers
	2. Available Resources	The level of resources dedicated to support the innovation grant, including money, training, education, physical space, staffing, and time
	3. Access to Knowledge & Information	Guidance and direction (including training) are accessible to facilitate the understanding of related concepts and the use of product
	Networks & Communications	Formal and informal relationships, networks, and interactions within and across structural, professional, or other [Inner Setting] boundaries. Include statements about general networking, communication, and relationships in the organization, such as descriptions of meetings, email groups, or other methods of keeping people connected and informed, and statements related to team formation, quality, and functioning
	User needs and resources	Consideration of the needs and resources of the target group (could be the needs of patients or the needs of clinicians)
Innovation Characteristics	Innovation Source	Perception of key stakeholders about how the template or the digital resource navigator was developed
	Evidence Strength & Quality	Stakeholders’ perceptions of the quality and validity of evidence supporting the belief that the intervention will have desired outcomes. Examples of evidence include peer-reviewed publication, reports, anecdote, and community feedback
	Relative Advantage	Stakeholders’ perception of the advantage of implementing the innovation versus an alternative solution Value-based payer agnostic care or the digital resource navigator is better or worse than other innovations or current practice
	Adaptability	The degree to which the innovation can be adapted, tailored, refined, or reinvented to meet needs in Orange County
	Trialability	The ability to test the innovation on a small scale in Orange County, and to be able to reverse course (undo implementation) if warranted
	Complexity	Developing a template for value-based payment contracts that promote agnostic care, and developing a digital resource navigator in the context of an innovation grant is complicated, which may be reflected by its scope and/or the nature and number of connections and steps
	Design Quality and Packaging	The innovation is well-designed and packaged, including how it is assembled, bundled, and presented. This is more applicable to the digital resource navigator.
Outer Setting	Cosmopolitanism	Networks and relationships between [the Inner Setting] and entities in [the Outer Setting] For example, spanning of boundaries between networks (e.g., health plans, payers, MHSOAC) and active participation between groups that may impact the implementation of value-based payor agnostic care or development of a digital resource navigator
	COVID-19	The effect of COVID-19 on innovation planning and development
	External Policies and Incentives	Legislation, guidelines, regulations, criteria, recommendations from the government and other influential entities
Process	Reflecting & Evaluation	Quantitative and qualitative feedback about innovation progress such as the annual MHSA Innovation Report
	Engaging	Attracting and involving appropriate individuals in innovation planning and development through a combined strategy of social marketing, education, role modeling, training, and other similar activities
	Planning	The degree to which a scheme or method of behavior and tasks for innovation development and planning is developed in advance, and the quality of those schemes or methods
Individual Characteristics	Knowledge, attitudes & beliefs about the Innovation	Individual attitudes toward and value placed on the template or product as well as familiarity with facts, truths, and principles related to development

Note. Definitions were adapted from the original CFIR definitions (https://cfirguide.org)

Researchers have used CFIR to understand complicated system transformation efforts within organizations and health care systems [20–22]. For example, Kilaru et al. (2022) interviewed regulators and health care agency leaders about the all-payor global budget system in Maryland; their analyses using CFIR revealed factors that facilitated the design, implementation, and sustainability of system transformation efforts, such as clear and reasonable expectations, the appropriate amount of autonomy within the global budget, close communication, actionable data, and shared commitment and readiness for change. As such, CFIR has demonstrated its applicability when evaluating system transformation in different settings.

### Evaluation context and aims

The BHST Innovation Project, approved by the Mental Health Services Oversight and Accountability Commission (MHSOAC), is a five-year Mental Health Services Act (MHSA) Project with a total budget of approximately $18 million. California uses innovation projects as part of their Mental Health Services Act (MHSA) program to provide resources for mental health. The goal of Orange County’s BHST Innovation Project is a system transformation effort to enable access to behavioral health services regardless of insurance status, insurance type, and/or level of clinical need. Specifically, this project included two parts:

1. Part 1: leveraging a value-based contract to align legal, fiscal, and regulatory requirements to improve the quality of behavioral health services, and implementing payor-agnostic care to improve access to care; and.
2. Part 2: developing a digital resource navigator to improve resource sharing and behavioral health service navigation.

The aim of this paper is to use the CFIR framework to evaluate facilitators of and barriers to the success of a behavioral health system transformation project. This paper fills a gap in knowledge by sharing learnings from the early stages of innovation in behavioral health payment and care and organizing these learnings in the CFIR model to promote their application to other projects. Although many systems are exploring such models, the learnings in county behavioral health settings from such explorations are too rarely shared. Moving towards value-based payor agnostic behavioral health care (Part 1) and improving access to information about care (Part 2) can help alleviate the unmet need within the County behavioral health system.

## Method

### Participants

As part of an evaluation of the BHST Innovation Project, 29 individuals participated in key informant interviews between May and August 2022. Participant information is available in Table 2. Seven individuals who had leadership roles at the County or a participating health care agency (L) participated in interviews that included both Part 1 and Part 2. Staff who only have knowledge about one part of the project participated in part-specific interviews, including eight contracted partners in Part 1 (CP1), four contracted partners in Part 2 (CP2), three community members and County stakeholders in Part 1 (CS1), and seven community members and County stakeholders in Part 2 (CS2). These interviewees were recruited due to their knowledge and involvement in the BHST Innovation Project.

**Table 2:** Participant information

Project Role	Total	Part 1. Develop a Template for Value-Based Contracts that promote payor-agnostic care	Part 2. Develop a digital resource navigator
County or Health Care Agency Leaders	7^a^	7^a^	7^a^
Contracted Partners	12	8	4
Community Members and County Stakeholders	10	3	7

Note. *N* = 29. ^a^County or health care agency leaders completed interviews that discussed both Part 1 and Part 2

The Consolidated Framework for Implementation Research (CFIR) was selected to guide the evaluation of both Part 1 and Part 2 in a consistent and systematic way.

### Data collection

Our institutional review board deemed that this work was exempt from human participant research approval (University of California, Irvine Institutional Review Board (IRB)# #20,195,406). All participants provided verbal consent prior to participating in the interviews.

We developed a semi-structured interview guide based on relevant constructs from the CFIR model (Damschroder et al., 2009). The interview guide included a set of general questions for all interviewees with additional tailored questions for interviewees with different project roles (e.g., CP1, CP2, CS1, CS2, L). Interview guides are available in the supplementary material (Supplement 1). Each interview question was anchored to a CFIR construct. All interviewers had expertise in program evaluation or implementation science (DS, RV, SMS). Interviews were a mix of one interviewer, two interviewers, and two interviewers and a notetaker. Each interview lasted approximately 30 to 60 min. A total of 29 interviews were conducted, auto-transcribed by Zoom, and then the transcripts were verified and cleaned by the evaluation team.

### Data analyses

We conducted thematic analyses following Braun and Clarke’s recommendation (2006). We outline how we follow their 6 proposed phases of analysis below.

#### Phase 1–2 (being familiar with data and initial coding)

XZ and RV were both trained in the CFIR framework, qualitative coding best practices, and use of the coding software (ATLAS.ti, version 22) prior to conducting data analyses. All data cleaning and analyses were completed using ATLAS.ti. We developed an initial draft of the codebook with adapted definitions of the CFIR constructs (Table 1). We used the five broad CFIR domains (intervention characteristics, outer setting, inner setting, characteristics of individuals, and process) and identified relevant subdomains. We completed initial coding, adapted the general CFIR definitions to be project-specific, and added a subdomain (i.e., COVID-19 as a factor in the outer setting). A total of 29 codes derived from the CFIR, including CFIR domains and subdomains, were included in the final codebook. Adapted definitions of the CFIR codes for both Part 1 and Part 2 of the project are presented in Table 2.

### Phase 3–5 (searching for, reviewing, defining, and naming themes)

XZ and RV double coded all transcripts. Initial percentages of agreement between two coders at the transcript level ranged from 46 to 74%. XZ and RV met weekly to review discrepancies and discuss revisions of the codebook (e.g., clarification of domain and subdomain definitions, addition of relevant subdomains). Coding was discussed during weekly team meetings to support consistency and resolve any discrepancies. These meetings were attended by the two coders and two other members of our research team (DS and SMS). Through discussion, final codes were decided for any discrepancies. Thus, our codes used for data analysis were codes with initial agreement or codes with discrepancies resolved through discussion with the broader research team.

We used ATLAS.ti software to calculate the frequency of the codes by CFIR domain and project aspect (Part 1 vs. Part 2) to obtain an overview of code distribution. This allowed an initial overview of codes and identification of which codes were more common for Part 1 and/or Part 2. We followed best practices in qualitative analyses mentioned by Braun and Clarke (2006) and constructed salient themes that “capture something important about the data in relation to the research and represents some level of patterned response or meaning within the data set.”

### Phase 6 (locating exemplars and producing the report)

XZ, RV, and SMS engaged in documenting the themes described in this paper. XZ built a narrative of the data and selected illustrative example quotes under each theme. XZ labeled individual participants; for example, an example quote from the first contracted partner in Part 1 (CP1) was labeled “CP1.1”. RV and SMS reviewed the themes and examples and provided feedback.

## Results

Guided by CFIR, we examined facilitators and barriers related to behavioral health care system transformation efforts in Orange County separately for each of the two parts: (Part 1) developing a template for value-based payment contracts that promote payor-agnostic care, and (Part 2) creating a digital resource navigator. Overall, five themes were identified from the key informant interviews including (1) aligning goals and values (2) assessing and addressing fit, (3) fostering partnership and engagement, (4) being aware of implementation contexts, and (5) promoting communication. In Table 3, we presented barriers and facilitators along with their CFIR domains related to each of the five themes. Different barriers and facilitators were identified for Part 1 and Part 2. Some barriers in the outer setting, such as changing state guidelines and priorities and fostering partnerships with private and nonprofit sectors, were unique to developing a template for value-based contracts that move toward payor-agnostic care. Engaging diverse communities to inform the design and content, mostly innovation characteristics, was a key facilitator for developing the digital resource navigator.

**Table 3:** Facilitators of and Barriers to Development of a Template for Value-Based Contracts that Promote Payor-Agnostic Care and Development of a Digital Resource Navigator

Theme	Facilitators and Barriers	CFIR domain	Part 1: Develop a template for Value-Based Contracts that promote Payor-Agnostic Care	Part 2: Develop a Digital Resource Navigator
Aligning goals and values	*Facilitator*: Alignment of values and shared enthusiasm *Barrier*: Misalignment of current goals and visions	inner setting, compatibility	x	x
	*Facilitator*: Leveraged strong management and leadership in the inner setting as internal champions can help align vision and goals	inner setting, leadership	x	
	*Facilitator*: Clearly communicated the relative advantage of the innovation to align goals	innovation characteristics, relative advantage		x
Assessing and addressing fit	*Barrier*: Lack of fit with existing health care system infrastructure, such as incentives for commercial plans and private companies	outer setting, external policies, and incentives	x	
Foster partnership and engagement	*Facilitator*: Partnering with philanthropic organizations to obtain funding that aligns with the project mission to facilitate moving the County behavioral health system towards payor-agnostic care	process, engaging	x	
	*Facilitator*: Successful outreach efforts to community organizations facilitated the process of gathering feedback from community members and raising awareness about the digital resource navigator	innovation characteristics, innovation source		x
	*Barrier*: COVID-19 disrupted partnership building and community engagement plans	outer setting, COVID-19	x	x
Being aware of implementation contexts	*Barrier*: Staff and leadership turnover, staffing shortage	inner setting, available resources	x	
	*Barrier*: Health care agencies had to shift their priorities due to COVID-19 related disruptions	inner setting, relative priority	x	
	*Barrier*: State-level policies and the current CA health care infrastructure, such as the “carve-out”, led to challenges in developing a value-based contract and moving towards payor-agnostic care	outer setting, cosmopolitanism	x	
Promoting transparent and efficient communication	*Barrier*: Lack of transparent communication led to a lack of shared understanding and trust within the project team	inner setting, network and communication	x	
	*Facilitator*: Proactive and transparent communication helped build trusting and collaborative relationships within the project team	inner setting, network and communication		x
	*Barrier*: Using academic and technical jargon was a barrier to communicating with community members and lay workforce	inner setting, network and communication	x	
	*Facilitator*: Routine check-ins and regular meetings with agenda	inner setting, network and communication		x

### Part 1: Develop a Template for Value-Based Payment Contracts That Promote Payor-Agnostic Care

Themes and example quotes for facilitators of and barriers to developing a template for value-based payment contracts that promote payor-agnostic care are presented in Table 4.

**Table 4:** Themes and Example Quotes for Facilitators and Barriers to Developing Value-Based Contracts that promote Payor-Agnostic Care

Themes	Facilitators and Barriers	CFIR domains	Example Quotes	
Aligning goals and values	*Facilitator*: Alignment of values and shared enthusiasm	inner setting, compatibility	From a clinician standpoint, it’s so much easier when a clinician can just treat the client and not have to worry about what type of insurance do they have, what can I not and what can I, and can I not do. What can or can they not receive for resources referrals.	
	*Barrier*: Misalignment of current goals and visions	inner setting, compatibility	I would say that I think that there has not been alignment and agreement on the focus or the vision or the purpose and it’s felt like a kind of ongoing debate in terms of whether we want a liberal or conservative interpretation of the Constitution. It’s just fundamental disagreement on how to come to what that approved proposal was and how lenient and open to interpretation that approval is, and therefore we have not been able to get on the same page.	
	*Facilitator*: Leveraged strong management and leadership in the inner setting as internal champions can help align vision and goals	inner setting, leadership	The previous Health Care Agency Director was a champion and then the previous Behavioral Health Director was a champion… just having external subcontractors moving it forward isn’t enough to be able to realize the full value of the planning project or kind of to be able to support what the resulting plan would be.	
Assessing and addressing fit	*Barrier*: Lack of fit with existing health care system infrastructure, such as incentives for commercial plans and private companies	outer setting, external policies, and incentives	It’s really hard to bring all the insurance companies to the table and say, ‘hey forget your profits, let’s just provide services at any cost’… the number one obstacle is getting those people to the table and questioning their profit level.	
Foster partnership and engagement	*Facilitator*: Partnering with community and philanthropy organizations	process, engaging	Bring philanthropy to the table as well, because we really have a lot of wealth in our county. Philanthropy and some sort of a fund that accrues good interest, and we could utilize that as a stopgap between someone who doesn’t have payment and someone who does.	
	*Barrier*: COVID-19 disrupted partnership building and community engagement plans	outer setting, COVID-19	We also had COVID, a lot of that [collaboration] got disrupted.	
Being aware of implementation contexts	*Barrier*: Staff and leadership turnover, staffing shortage	inner setting, available resources	When there was turnover… that’s where kind of some of the thread may have gotten lost a little bit…. The focus of [value-based contracting] …was diminished…	
	*Barrier*: Health care agencies had to shift their priorities due to COVID-19 related disruptions	inner setting, relative priority	There’s been a huge amount of distraction from the focus on COVID, and COVID response and vaccine, and response to COVID response… the transformation in the Community that happened from the CARES [The Coronavirus Aid, Relief, and Economic Security] Act dollars and other things getting poured in. It really took up a whole lot of time and space from elected officials to County staff to providers to take that in beyond what the usual kind of extravaganza of MHSA funding does every year. It really took all of that and poured gasoline on that fire, so it really changed the capacity of folks to engage in the work that we were trying to do.	
	*Barrier*: State-level policies and the current CA health care infrastructure, such as the “carve-out”, led to challenges to developing a value-based contract and moving towards payor-agnostic care	outer setting, cosmopolitanism	In California, as long as the carve-out remains, there’re only so many levers you can pull.	
Promoting transparent and efficient communication	*Barrier*: Lack of transparent communication led to a lack of shared understanding and trust within the project team	inner setting, networks and communication	It’s very important to me about communication, and I have to be honest, right now, it’s a lack of communication with the Community.	
	*Barrier*: Using academic and technical jargon was a barrier to communicating with community members and lay workforce	inner setting, networks and communication	We are not baking a cake, so stop using the word measure. This is technical jargon; this is behind-the-scenes jargon. If you’re giving this to the Community, it should be as simple as I’m talking to you right now’	

#### Aligning goals and values

Despite shared enthusiasm about value-based payment models that promote payor-agnostic care, misalignment in vision and scope was a barrier (inner setting, compatibility). For example, CP1.1 shared their excitement for increasing access to care and expressed a desire for payor-agnostic care (e.g., “From a clinician standpoint, it’s so much easier when a clinician can just treat the client and not have to worry about what type of insurance do they have, what can I not and what can I, and can I not do. What can or can they not receive for resources referrals”). Despite the shared enthusiasm among contracted partners and County health care agency leaders about increasing access to care via payor-agnostic care, perceptions of vision and scope of the contract varied, posing barriers in the inner setting. A County health care agency leader (L.1) described this barrier: “I would say that I think that there has not been alignment and agreement on the focus or the vision or the purpose and it’s felt like a kind of ongoing debate in terms of whether we want a liberal or conservative interpretation of the Constitution. It’s just fundamental disagreement on how to come to what that approved proposal was and how lenient and open to interpretation that approval is, and therefore we have not been able to get on the same page.” Confusion about the scope of the current project vision (e.g., L.2: “How it’s going to happen, I have no idea”) and skepticism about its feasibility (e.g., L.1: “Payor-agnostic… too ambitious and it’s certainly not doable or feasible in the time left on the project”) were barriers that tempered the enthusiasm for the project. Leveraging strong management and leadership (inner setting, leadership) as internal champions facilitated the process of aligning visions within the organization (e.g., “… the previous Health Care Agency Director was a champion and then the previous Behavioral Health Director was a champion… just having external subcontractors moving it forward isn’t enough to be able to realize the full value of the planning project or… to be able to support what the resulting plan would be.”).

#### Assessing and addressing fit

Lack of fit with existing health care system infrastructure was identified as a barrier to developing a template for value-based payment contracts that move toward payor-agnostic care. County health care agency leaders mentioned that private and public payors had different priorities and incentives within their organizations (outer setting, external policies and incentives). When describing challenges of bringing the private sector to the table, county health care agency leaders (L.2, L.4) used words such as “profit” and “return on investment.” L.2 described that a lack of incentives for commercial plans and private companies was a key challenge to engaging commercial payors: “It’s really hard to bring all the insurance companies to the table and say, ‘hey forget your profits, let’s just provide services at any cost’… the number one obstacle is getting those people to the table and questioning their profit level”. In contrast, the public sector had a bigger focus on compliance. For example, certified public expenditures (CPE) in the public sector were described as very specific (L.3). As described by L.5, the lack of flexibility of CPE suggested a poor fit between the value-based contracting and public funding structure: “So, I think for the Medi-Cal payment, I don’t think that we’re there, and we can’t gift public funds as a reward or an incentive to providers. It’s not laid out there. I know there are conversations at the state, but I think we’re [Orange County] so far ahead, as I understand it, and we don’t have the ability to just pay people extra, let them keep things that …there’s not a cost to it.”

#### Foster partnership

Strong cross-sector partnerships facilitated the process of braiding different funding streams. The importance of private-public payor partnership was recognized, especially related to factors in the outer setting. For instance, staff members and community stakeholders reported successful buy-in from commercial plans (outer setting, cosmopolitanism). CP1.2, CP1.3, and CS1.1 mentioned Kaiser Permanente as an example. CP1.2 stated: “Some of them [Commercial Plans] were already there. I mean Kaiser was a very early participant. They were an investor…They’re a big component of the… ecosystem, and they’re very much there”). This indicated a clear need for more efforts to facilitate the partnership with private insurance companies. CP1.2 also shared that their team’s cross-sector background and expertise facilitated establishing relationships and building cross-sector partnerships: “I come from a place of cross-sector, cross-organizational collaboration, and I think we can only improve what we’re doing if we learn what’s happening in other people’s backyards in like… how hard their jobs are”. Additionally, L.3 mentioned partnering with philanthropic organizations to obtain funding that aligns with the project mission (i.e., being able to serve everyone regardless of insurance status and clinical needs) could facilitate moving the County behavioral health system towards payor-agnostic care: “bring philanthropy to the table as well, because we really have a lot of wealth in our county. Philanthropy and some sort of a fund that accrues good interest, and we could utilize that as a stopgap between someone who doesn’t have payment and someone who does.” Participants with different roles on the project (CP1.1, CP1.3, L.3, L.4, CS1.2) described the COVID-19 global pandemic as disruptive to partnership relationship building and capacity of community members and staff members (outer setting, COVID-19). For example, CP1.3 stated: “We also had COVID, a lot of that [collaboration] got disrupted.”

#### Being aware of implementation contexts

A multitude of barriers influenced implementation contexts, including workforce challenges, the impact of COVID-19, and state-level policies. Workforce challenges were related to barriers in both inner and outer settings. Staff turnover and limited time and bandwidth were noted challenges in the inner setting and oftentimes led to de-prioritization of value-based contracting over other initiatives, particularly within the context of various state regulations (outer setting, external incentives and policies). For example, CP1.3 stated: “When there was turnover… that’s where kind of some of the thread may have gotten lost a little bit…. the focus of [value-based contracting] …was diminished…”. Relatedly, health care agencies had to shift their priorities due to COVID-19 related disruptions (e.g., CP1.3: “COVID changed things…there’s been a huge amount of distraction from the focus on COVID, and COVID response and vaccine, and response to COVID response… the transformation in the Community that happened from the CARES [The Coronavirus Aid, Relief, and Economic Security] Act dollars and other things getting poured in. It really took up a whole lot of time and space from elected officials to County staff to providers to take that in beyond what the usual kind of extravaganza of MHSA funding does every year. It really took all of that and poured gasoline on that fire, so it really changed the capacity of folks to engage in the work that we were trying to do.”). In addition to COVID-19 related disruptions and initiatives, state-level policies and the current CA health care infrastructure, such as the “carve-out” (the separation of mental health and substance use treatment services from the broader health care system), were mentioned by staff members and community stakeholders as barriers (CS1.3, CP1.2, CP1.3, CP1.4). CP1.2 described: “…in California, as long as the carve-out remains, there’re only so many levers you can pull.”

#### Promoting transparent and efficient communication

The complexity of developing a template for value-based contracts requires transparent and efficient communication. The need for promoting communication was identified as a theme related to building trust and relationships within a team (inner setting, networks and communication) and cultivating external partnerships (outer setting, cosmopolitism). A lack of transparent communication was identified as a barrier in both inner and outer settings. For example, L.2 described that the lack of communication could lead to a lack of shared understanding and trust within the project team (inner setting). L.4 mentioned that open communication with the state about the project progress was necessary to obtain state support and guidance (outer setting). To enhance communication among diverse stakeholders, the need for tailoring communication styles was mentioned. Participants (CP1.5, CS1.2, CS1.3) implied that using academic and technical jargon could be a barrier to communicating with community members and the lay workforce. For example, CS1.2 stated: “I looked at the summary of the survey and I said ‘…we are not baking a cake, so stop using the word measure. This is technical jargon; this is behind-the-scenes jargon. If you’re giving this to the Community, it should be as simple as I’m talking to you right now’.

### Part 2: develop a Digital Resource Navigator (OC Navigator)

Themes and example quotes for facilitators of and barriers to developing a digital resource navigator to improve resource sharing and behavioral health service navigation are presented in Table 5.

**Table 5:** Quotes for Facilitators and Barriers to Developing a Digital Resource Navigator

Themes	Facilitators and Barriers	CFIR domains	Example Quotes
Aligning goals and values	*Facilitator*: Alignment of values and shared enthusiasm	inner setting, compatibility	It’s like knowing that the people that I’m interfacing with, the people that I’m like bothering and requesting meetings for, they all have the same … we all share the same goal of like wanting to help people, and improve services, and improve access to services
	*Barrier*: Misalignment of current goals and visions	inner setting, compatibility	We got a lot of comments and there’s a lot of chatter in the Community about is this [digital resource navigator] a waste of money because it’s a redundant resource?
	*Facilitator*: Clearly communicated the relative advantage of the innovation to align goals	innovation characteristics, relative advantage	I would say it’s changed workflows in terms of centralizing and digitizing what used to be manual paper notes, post-it notes, some documentation here, some documentation there. They’ve digitized and, in some cases, automated a lot of the workflows for our telephone-based navigation line
Foster partnership and engagement	*Facilitator*: Successful outreach efforts to community organizations facilitated the process of gathering feedback from community members and raising awareness about the digital resource navigator	innovation characteristics, innovation source	we have been successful in this sense where we’ve been talking to organizations and collaboratives and coalitions. Our pitch has been… give us your resource directory. We will highlight it on the website [the digital resource navigator]. You can correct it. We’ll tell you who authored it. We will give you a link that highlights your website, and these resource directories on the site and just let us help us help you, and then do your job better.
	*Barrier*: COVID-19 disrupted partnership building and community engagement plans.	outer setting, COVID-19	At the beginning of the project, it was definitely like come one come all because we were getting started, it was COVID and really hard to engage
Promoting transparent and efficient communication	*Facilitator*: Proactive and transparent communication helped build trusting and collaborative relationships within the project team	inner setting, network and communication	There’s a very wide level of trust within everybody in the team to making the best decisions as possible for the project and for staff, so less checks and balances there, and more like this is what the Community wants. This is what we’re going to try to do as much as possible.
	*Facilitator*: Routine check-in and regular meetings with agenda	inner setting, network and communication	On Thursday, we talked about how we are going to do Friday’s meeting. What approach are we going to use to try to get input? We sent the agenda out. [Staff member] asked for agenda items prior to the meeting, so we keep trying different things.

#### Aligning goals and values

Shared enthusiasm was a facilitator for the development of the digital resource navigator. Specifically, its clear fit with County values and workflows in the inner setting and its relative advantages (innovation characteristics) contributed to the shared enthusiasm. Participants with different roles shared the same goal of improving existing workflows and increasing patient care in Orange County (inner setting, compatibility). As described by CP2.1: “It’s like knowing that the people that I’m interfacing with, the people that I’m like bothering and requesting meetings for, they all have the same like… we all share the same goal of like wanting to help people, and improve services, and improve access to services.” Multiple community stakeholders (CS2.1, CS2.5) described the strong fit between the digital resource navigator and the County’s equity-driven values. For instance, CS2.1 described the digital resource navigator as in line with the equity-driven values of the County: “One of our [Orange County Health Care Agency’s] key focuses was decreasing inequity, increasing equity… this is going to the next step on bringing more resources… I would say absolutely [the digital resource navigator] fits with Orange County’s values and workflows”. In addition to the clear fit between the digital resource navigator and County values, the strong fit between the digital resource navigator and existing workflows also contributed to enthusiasm among providers and community members. L.2 described that the digital resource navigator improved the efficiency of day-to-day tasks of County staff: “It’s helping the workflows [at OCHCA] be more efficient and cut out the extra unnecessary steps. It definitely is working to improve the system at the County.” Despite its fit into the larger values and workflows of Orange County Health Care Agency, not everyone deemed the digital resource navigator as a current need. For example, CP2.2 raised the question about whether the digital resource navigator was a redundant resource in the community: “We got a lot of comments and there’s a lot of chatter in the Community about is this [digital resource navigator] a waste of money because it’s a redundant resource?”.

The clear relative advantages (innovation characteristics) of the digital resource navigator contributed to shared enthusiasm among stakeholders and contracted partners. One relative advantage (innovation characteristics) was implementing a more centralized information-sharing, compared to the traditional paper-pencil format and other existing online tools. For example, L.1 stated: **“**I would say it’s changed workflows in terms of centralizing and digitizing what used to be manual paper notes, post-it notes, some documentation here, some documentation there. They’ve digitized and in some cases automated a lot of the workflows for our telephone-based navigation line”. The increased efficiency in referral workflow was described as another relative advantage of the digital resource navigator. CP2.2 stated: “…you get to a point [when using other applications to find behavioral health resources] and you’d be stuck, and you just have to call the agency. You might have a list of twenty agencies, and you have to call them all before you can get some real basic information. But ours has… the way that they’re [the digital resource navigator] setting up the information cards. They give a lot of information, and then it just seems to be more robust and user-friendly.”

#### Fostering Engagement

Community engagement was identified as a process factor that facilitated the development and improvement of the digital resource navigator. Outreach efforts to community organizations facilitated the process of gathering feedback from community members and raising awareness about the digital resource navigator, as described by a community stakeholder (CS2.2) and multiple contracted partners (e.g., CP2.1, CP2.2, CP2.3). CP2.2 described: “… we have been successful in this sense where we’ve been talking to organizations and collaboratives and coalitions. Our pitch has been… give us your resource directory. We will highlight it on the website [the digital resource navigator]. You can correct it. We’ll tell you who authored it. We will give you a link that highlights your website, and these resource directories on the site and just let us help us help you, and then do your job better.” CP2.3 and CS2.2 identified connections with external networks as an important innovation source (e.g., National Council of Negro Women, National Alliance on Mental Illness (NAMI)) because they increased the team’s knowledge base about available resources and community needs. For example, CS2.2 described that conversations with NAMI informed the design, content, and implementation of the digital resource navigator: “So it was really great to be able to sit with [the technology vendor team] and have them ask sort of what [NAMI’s] vision was for the database and search platform to be, how we wanted it implemented, and just even how it looks so it’s not so scary” (innovation source, innovation characteristics).

Engaging community members, such as individuals with lived experiences and from marginalized groups, facilitated the improvement of design features. CP2.4 recommended leveraging community champions as a way of reaching historically marginalized groups: “Have [County leaders] help us identify champions in the community that might be good representatives for different sorts of things. So that kind of got us started. I remember a couple of years ago… they said, ‘this group is working on this, and this group is working on that’. They kind of helped with some initial introductions.” Despite the support from champions, engaging community members was especially challenging in the context of the COVID-19 global pandemic. COVID-19 lockdowns disrupted engagement with consumers who needed or preferred in-person engagement, as described by CP2.1: “At the beginning of the project, it was definitely like come one come all because we were getting started, it was COVID and really hard to engage.” (COVID, outer setting).

Lack of connections with historically marginalized communities was a barrier to curating multilinguistic and culturally relevant content on the digital resource navigator. The contracted partner’s team (e.g., CP2.3) commented on the earlier challenges of connecting with the Spanish-speaking community: “…not having been able to connect with more Spanish-speaking populations than we have. Along with that, having all the other languages that we represent on the Navigator… Not really having that much direct access to folks in that community”. Additionally, CP2.2 described that community engagement could have been more helpful in the early planning process of the project: “…we recently had a couple work groups with… a group of Latinas. They were Spanish-speaking only and they actually had a lot of suggestions because they actually have their own Facebook group, but these are the conversations I wish we’d had a little bit earlier. But better late than never.” In addition to engaging Latinx and Spanish-speaking populations through Spanish work groups (CS2.3, CS2.4), multiple participants (CP2.2, CP2.3, CS2.3, CS2.5) also mentioned that effective marketing and outreach efforts might facilitate the process of connecting with other historically underrepresented groups, such as veteran communities, faith-based organizations, individuals with special needs, and Korean and Chinese communities.

#### Promoting transparent and efficient communication

Transparent and open communication between County, contracted partners, and community partners was the most highlighted facilitator. CP2.1 described that a trusting relationship allowed more time and resources to be allocated to innovation development and community engagement: “There’s a very wide level of trust within everybody in the team to making the best decisions as possible for the project and for staff, so less checks and balances there, and more like this is what the Community wants. This is what we’re going to try to do as much as possible.” Multiple participants across different roles on the project (CP2.1, CP2.4, L.6) expressed that they were able to build trusting and collaborative relationships with proactive communication. Regularly scheduled meetings with clear agenda items, openness to feedback, active incorporation of feedback from community members, and discussions about specific design features and usefulness of the content improved design and feature development of the digital resource navigator. For example, CP2.2 described the process of engaging community co-chairs in the decision making around the need for broader community outreach, “for short things we run it by them [community co-chairs] and sort of get their temperature about if we should ask a broader group. Then, of course, we asked the County…it’s not a perfect process…and it’s iterative but we try our best….”.

## Discussion

This paper fills a literature gap by disseminating insights garnered from the initial phases of innovation in behavioral health payment and care. Applying the CFIR framework, we identified important facilitators of and barriers to Orange County’s behavioral health system transformation efforts under several key themes in the context of an innovation project. Specifically, aligning goals and values, fostering engagement and partnership, and promoting communication were all highlighted as important factors related to developing a template for value-based contracts that promote payor-agnostic care (Part 1) and creating a digital resource navigator (Part 2). Changing state guidelines and priorities, different incentive structures within the US health care system, and difficulties in braiding public and private funds (e.g., private insurance companies, philanthropic organizations) were unique barriers to developing a template for value-based contracts that promote payor-agnostic care. Leveraging diverse communities to inform the design and content of the digital platform, mostly in the domain of innovation characteristics, was a facilitator of creating the resource navigator.

### Part 1. Develop a template for value-based payment contracts that promote payor-agnostic care

Misalignment in incentives, values, and goals posed barriers to developing a template for value-based contracts. Value-based payment models and payor-agnostic care are a disruption to the status quo of fee-for-service predominance and service fragmentation by payor source. Thus, the change to value-based payments could be perceived as adding regulatory and financial risks for both public and private sectors. Similar to challenges identified in our study, in a study of value-based care for substance use disorder treatment, researchers identified providers’ buy-in to value-based concepts as a key workforce challenge [11]. Additionally, we found developing a template for value-based contracts was perceived as a lower priority compared to other organizational and state initiatives due to limited agency bandwidth and competing priorities during the COVID-19 global pandemic. This was not unique in this implementation context. Delayed transition to value-based payments has been common in many hospital settings. For example, the Centers for Medicare and Medicaid Services (CMS) stopped accepting applications for new Accountable Care Organizations (a type of value-based contract) in 2021 [23].

Providing financial incentives may increase the acceptability of value-based contracts that promote payor-agnostic care. Existing financial strategies often rely on public funds, such as increased pay-for-service, grants, and cost-sharing, which may not be a sustainable and scalable solution [1]. Braiding public and private funding streams may help change the current incentive structure and provide sustainable resources for implementing value-based payment and payor-agnostic care. Funding from philanthropic organizations may be a potential funding source to fill some gaps in the behavioral healthcare sphere, and venture capital firms are getting involved in creating new innovations that may increase access to behavioral health services [24]. With both private and public sectors around the table, long-term cost-saving potentials of a value-based contract may act as a shared motive towards creating momentum that is needed to facilitate system transformation efforts [25].

#### Part 2: develop a Digital Resource Navigator (OC Navigator)

Our work furthers the understanding of factors influencing the development of a digital resource navigator. We find that aligning goals and values, fostering engagement, and promoting transparent and efficient communication were important to the development and implementation of a digital resource navigator in Orange County. In our analyses, the perceived compatibility between the digital resource navigator and the extent to which it improved the current referral process workflow impacted the enthusiasm about the digital resource navigator. Consistent with our findings, past research has also found that providers were more likely to implement a technology-enabled tool when the tool could fit into or improve the existing workflow [26, 27]. Our data also revealed that some interviewees did not deem the digital resource navigator as a priority. Similarly, Zhao et al. (2022) found that some providers perceived implementing a technology-enabled tool as a low priority, especially considering that there were limited resources in their organizations.

Inclusive design, communication and engagement strategies allowed the contracted partners to better align a digital resource navigator with the needs and priorities of the diverse communities in Orange County. In particular, it is important to understand the unique needs for information and resources among marginalized and underrepresented populations (e.g., monolingual communities, faith-based organizations) [28]. The design of technology-enabled behavioral health tools, including the digital resource navigator that was evaluated in this study, needs feedback from diverse community stakeholders. In our analyses, staff members and community champions shared their wishes for engaging diverse community members *early* in the iterative design process of the digital resource navigator. This cross-sector collaboration process requires inclusive communication strategies, as contracted partners, community stakeholders, and academic evaluators often speak different languages and have different levels of technological understanding. It was highlighted in our data that leveraging connections and knowledge of community champions not only facilitated communication but also outreach to the broader community. However, it is also worth noting that centering community voice and consistent outreach activities often required additional staff time and bandwidth, which could be challenging within the constraints of the County’s resources and regulations. Additionally, ongoing tailoring and adaptation of existing resources on the digital resource navigator are necessary. For example, community members shared frustration when resources on the platform were outdated. The process of sustaining timely updates of platform content may be costly and create workforce challenges.

### Limitations

As found in the current study, external state policies and financial incentives in the outer setting were barriers to the acceptance of value-based and payor-agnostic care; although, government initiatives, such as California Advancing and Innovating Medi-Cal (CalAIM), could also facilitate the process. Attitudes and approaches towards system transformation may differ by state due to different state initiatives. It is important to note this project, along with a few mentioned past studies [8, 9] relied on public funding, which could be even more limited in lower-resourced states and counties. This project was conducted in the state of California, which has more resources than other states, as evidenced by higher income and higher GDP. Substantial variations in resources (e.g., provider availability, funding) at the County and state level may also impact agency bandwidth to pilot value-based payment contracts [25] and develop and sustain a digital resource navigator. Additionally, we did not conduct consumer interviews and observations. Consumer perspectives and outcomes can be particularly helpful in curating inclusive content and improving the user interface of the digital resource navigator. However, it is worth noting that we included a diverse group of interviewees, including staff members, leadership, and community stakeholders. Some of the interviewees worked closely with the community being served in this early stage of the grant. Additionally, the data were from an early stage (first 2.5 years) of a five-year grant-funded project in Orange County; some identified facilitators (e.g., shared enthusiasm about a new and exciting project) and barriers (e.g., COVID-19) may be related to the time point.

### Future directions

First, given the influence of external state policies, financial incentives, and County resources on the acceptability of value-based and payor-agnostic care, further investigation is needed to understand the impact of a specific County health care initiative on behavioral health care system transformation efforts. Comparative evaluation of barriers to and facilitators of various system transformation efforts across states and counties can provide valuable insights into policy implications and guide tailored support for local stakeholders. Second, centering the voices and experiences of consumers can be particularly helpful in ensuring the inclusivity of the content and design of the digital resource navigator. Collecting data from consumers through interviews and surveys can facilitate understanding the perceived usefulness and usability of the platform and content on the digital resource navigator.

## Conclusion

We analyzed 29 key informant interviews to provide insight into the barriers and facilitators related to County behavioral health system transformation in a state-funded project. Overall, aligning goals and values, fostering engagement and partnership, and promoting communication were important factors to consider when developing a template for value-based contracts that promote payor-agnostic care (Part 1) and developing a digital resource navigator (Part 2). Being aware of changing state guidelines and priorities, having cross-sector specialty knowledge about incentive structures in the public and private sectors, and braiding public and private funds were important to developing a template for value-based contracts that promote payor-agnostic care. Leveraging diverse communities to inform the design and content and incorporating their timely feedback was particularly important to the development of the resource navigator. As these insights were drawn from diverse perspectives within the County Behavioral Health system, we hope that our research will prove invaluable to similar transformation endeavors in the future.

### Supplementary Information

**Supplementary Material 1.**

## Data Availability

The data collected and analyzed for this study are not publicly available because this was not a requirement of the project’s funder, and the verbal consent process did not elicit participants’ consent for their data to be publicly shared.
