# Supplementary material for: Facilitators of and barriers to County Behavioral Health System Transformation and Innovation: an interview study
Source: BMC Health Serv Res. 2024 May 9;24:604. doi: 10.1186/s12913-024-11041-9 (PMC11080221; doi:10.1186/s12913-024-11041-9)
Supplement: Supplementary file 1 — Supplementary Material 1. [file 12913_2024_11041_MOESM1_ESM.docx]

**Key Informant Interview Guide**

**Interview Guide for Interviews with Contracted Partners, Community Members and County Stakeholders in Part 1 (Develop A Template for Value-Based Contracts That Promote Payor-Agnostic Care)**

*Thank you for taking the time to meet with us today about the Behavioral Health System Transformation Project. [Similar to when we spoke six months ago,] The purpose of this interview is to understand a range of factors and processes that have or might impact the success of the first part of the Behavioral Health System Transformation Project that has to do with the creating a value-based payer agnostic system in Orange County. This interview should take approximately 60 minutes. We would like to record the interview so the Evaluation Team can review it later. We are here to listen to your perspectives, so we will not be sharing our opinions. Do you have any questions before we begin?*

1. Can you tell me a little about who you are and what your affiliation/background is?
   1. PROBE: How long have you been working in the behavioral health field?
   2. PROBE: What is your role on the BHST project?
2. When people talk about Behavioral Health System Transformation in Orange County, what does that mean to you?
   1. PROBE: What are the various goals, objectives, or programs related to BHST?
3. What was your initial vision for a payor agnostic value-based contracting model?? *(Innovation Characteristics – Innovation Source)*
   1. PROBE: What was the final product/result/outcome you wanted to achieve?
   2. PROBE: How did this change throughout the project?
4. Where are you on the path of your initial vision? What barriers/challenges do you foresee in achieving the vision?
5. To what extent are you aware of other efforts (i.e. state/federal) to create a payer agnostic system that uses Value Based Payment contracts and how did that inform your work? *(Outer Setting – Cosmopolitanism)*
   1. What information were you aware of or did you find important to gather while planning for a payer agnostic system that utilizes value-based contracts? How are you using this information to inform your work? *(Innovation Characteristics – Evidence Strength & Quality)*
   2. How does a value-based payer agnostic system compare to other ways to pay for Behavioral Health Services? What do you believe is the advantage of a value-based payer agnostic system in comparison to other payment systems? *(Innovation Characteristics – Relative Advantage)*
6. How did you determine what individuals and stakeholders needed to be involved in project planning?
   1. PROBE: How did you continue to engage those individuals?
7. What individuals and organizations have you reached out to as you planned and developed the framework for a value-based payer agnostic system? Can you discuss your work with them and how you have incorporated their input? *(Outer Setting – Cosmopolitanism)*
   1. PROBE: Stakeholders within Orange County and individuals or organizations outside of Orange County, perhaps even in different states
8. How did specific meeting formats [in the past project period] affect engagement, if at all? Did this differ across populations? *(Process - Engaging)*
9. Can you tell me about your efforts to better understand what is valuable to OC residents and stakeholders in the past project period as it relates to a payer agnostic value-based system? *(Inner Setting – Patient Needs & Resources)*
   1. PROBE: community members/consumers, clinicians, health plans, etc
   2. PROBE: What challenges did you face in using the community values to inform performance metrics and program standards? *(Inner Setting – Patient Needs & Resources)*
   3. PROBE: How are you getting information about your efforts back to the community?
10. How does a payer agnostic system that uses value based contracting fit with the values of consumers, clinicians, and health plans? How does it fit with currently established workflows (e.g., regulations, contracts, etc)? (*Inner Setting – Innovation Climate – Compatibility)*
11. Can you tell me about your approach to building a relationship with public and private payers? *(Inner Setting – Networks & Communications)*
12. Can you tell me about your working relationship with Mental Health Services Oversight and Accountability Commission (MHSOAC) as it relates to the BHST Innovation project? *(Inner Setting – Networks & Communications)*
13. Can you tell me about your working relationship with OCHCA as it relates to the BHST project? *(Inner Setting – Networks & Communications)*
    1. PROBE: Leadership, MHSA Innovations, other departments
    2. PROBE: What do you value most about your relationship with Orange County Health Care Agency (OCHCA)?
    3. PROBE: What advice or recommendations do you have to strengthen the collaboration with OCHCA?
    4. PROBE: Are there individuals or entities at HCA that you would like access to that you haven't been able to access at this time?
14. How, if at all, has leadership at the County supported the planning and eventual implementation of a payer agnostic system [this past project period]? Where, if at all, do you see areas where more County support is needed? *(Inner Setting – Readiness for Implementation)*
15. To what extent have you interfaced with BHST Part 2, and how, if at all, has it impacted the project? *(Inner Setting – Networks & Communications)*
16. With CalAIM payment reform becoming effective July 2023, how has BHST's work to structure a payer agnostic system that uses value-based contracting in Orange County been set up in a way that can be adapted and refined to fit CalAIM’s regulatory policies? *(Innovation Characteristics – Adaptability)*
17. Are there any barriers or challenges that we haven’t covered yet that you would like to tell me about? *(Process – Reflecting & Evaluating)*
18. Are there other thoughts and experiences regarding the BHST Innovation project that you would like to share? *(Process – Reflecting & Evaluating)*

**Interview Guide for Interviews with Contracted Partners and Community Members and County Stakeholders in Part 2 (Develop a Digital Resource Navigator)**

*Thank you for taking the time to meet with us today about the Behavioral Health System Transformation Project. The purpose of this interview is to understand a range of factors and processes that have or might impact the success of the second part of the Behavioral Health System Transformation Project that has to do with the Digital Resource Navigator (OC Navigator) in Orange County. This interview should take approximately 60 minutes. We would like to record the interview so the Evaluation Team can review it later. We are here to listen to your perspectives, so we will not be sharing our opinions. Your responses will remain anonymous. Do you have any questions before we begin?*

[TURN ON AUDIORECORDER]

1. Can you tell me a little about who you are, what your affiliation is, your role on the OC Navigator project, and how long you’ve been working on this project?
2. It’s clear that a lot of work has gone into the planning of the OC Navigator. Why do you think the OC Navigator is going to work?
   1. PROBE: What information have you found that shows whether or not the OC Navigator will be successful? *(Innovation Characteristics – Evidence Strength & Quality)*
      1. E.g., literature review, anecdotal evidence, etc.
   2. PROBE: How does the OC Navigator compare to other ways to find behavioral health resources in Orange County? *(Innovation Characteristics – Relative Advantage)*
3. To what extent are you aware of other efforts (i.e., state/federal) to build a consumer facing digital resource navigator and how did they influence the planning and design of the OC Navigator? *(Outer Setting – Cosmopolitanism)*
4. Can you tell me about your efforts to better understand what is valuable to OC residents and the County in the past project period as it relates to the OC Navigator *(Inner Setting – Patient Needs & Resources)*?
   1. PROBE: What have been some of the challenges around creating the OC Navigator while centering the community voice?
   2. PROBE: How are community priorities/concerns addressed?
   3. PROBE: How are you getting information back to the community?
5. How did you determine what individuals and stakeholders needed to be involved in OC Navigator planning and implementation? *(Process – Engaging)*
   1. PROBE: How did you continue to engage these individuals? *(Process – Engaging)*
6. What individuals and organizations have you reached out to as you planned and developed the OC Navigator? Can you discuss your work with them and how you have incorporated their input? *(Outer Setting – Cosmopolitanism)*
   1. PROBE: Stakeholders within Orange County and individuals outside of the county, perhaps even in different states.
7. How did specific meeting formats in this project period affect engagement, if at all? Did this differ across populations? *(Process – Engaging)*
8. How, if at all, has piloting the OC Navigator led to or informed changes or updates to the platform (e.g., front end and back end resources – OC Navigator, OC Links, CSU Bed board, etc)? *(Innovation Characteristics – Trialability)*
9. Can you tell me about your working relationship with Mental Health Services Oversight and Accountability Commission (MHSOAC) as it relates to the BHST project? *(Inner Setting – Networks and Communications)*
10. Can you tell me about your working relationship with Orange County Health Care Agency as it relates to the BHST project? *(Inner Setting – Networks and Communications)*
    1. PROBE: What do you value most about your relationship with Orange County Health Care Agency? *(Inner Setting – Networks and Communications)*
    2. PROBE: What advice or recommendations do you have to strengthen the collaboration with OCHCA? *(Inner Setting – Networks and Communications)*
11. Can you describe the way leadership at the County provided support for the planning and implementation of the OC Navigator? *(Inner Setting – Readiness for Implementation)*
    1. PROBE: Are there areas where more County support is needed? If so, what are they?
    2. PROBE: Who, if any, are the individuals or entities at OCHCA that you would like to build a relationship with/connect with that you haven’t been able to at this time? *(Inner Setting – Networks and Communications)*
12. Can you tell me about how the team composition and relationships/dynamics within [Contracted Partner Company 2] have influenced the project? *(Inner Setting – Networks & Communications)*
13. To what extent have you interfaced with BHST Part 1? And how, if at all, has it impacted the project?
14. Are there any barriers or challenges that we haven’t covered yet that you would like to tell me about? *(Process – Reflecting & Evaluating)*
15. Are there other thoughts and experiences regarding the OC Navigator or the BHST Innovation project that you would like to share?

**Interview Guide for Interviews with County Health Care Agency Leaders**

*Thank you for taking the time to meet with us today about the Behavioral Health System Transformation Project. The purpose of this interview is to understand a range of factors and processes that have or might impact the success of both parts of the Behavioral Health System Transformation Project that has to do with creating a payer agnostic value based contracting system and creating a Digital Resource Navigator (OC Navigator) in Orange County. This interview should take approximately 60 minutes. We would like to record the interview so the Evaluation Team can review it later. We are here to listen to your perspectives, so we will not be sharing our opinions. Do you have any questions before we begin?*

1. Can you tell me a little about who you are and what your affiliation/background is?
   1. PROBE: How long have you been working in the behavioral health field?
2. When people talk about Behavioral Health System Transformation in Orange County, what does that mean to you?
   1. PROBE: What are the various goals, objectives, or programs related to BHST?

Our role here at UCI is to evaluate two specific components of Innovation funding related to BHST. The first component relates to developing a payer agnostic value-based contracting model and the second relates to developing the OC Navigator.

Are you familiar with both components?

[If he says no – then change language below to reflect comments from Question #2]

[If he says yes]. As we move forward, we would like you to specifically think about these two components being funded through MHSA Innovation (payor agnostic/VBP payment reform and OC Navigator).

Payor agnostic/VBP reform:

1. What was your initial vision for a payor agnostic value-based contracting model? *(Innovation Characteristics – Innovation Source)*
   1. PROBE: What do you hope this project will achieve?
   2. PROBE: How did this change throughout the project?
2. What barriers/challenges do you foresee in achieving your vision for a payor agnostic value-based contracting model in Orange County? *(Characteristics of Individuals – Knowledge & Beliefs About Innovation)*
3. [Contracted partner 1] is leading the effort on payor agnostic/Value-Based Payment reform. How familiar are you with their approach? What are some of the strengths of their approach? What are some of the challenges?
4. How, if at all, has leadership at the County supported the planning and eventual implementation of a payor agnostic system that uses value-based contracting? Where, if at all, do you see areas where more support is needed? *(Inner Setting – Readiness for Implementation)*
5. How does a payer agnostic system that uses value based contracting fit with the values of OCHCA? How does it fit with currently established workflows (e.g., regulations, contracts, etc)? (*Inner Setting – Innovation Climate – Compatibility)*
6. Can you tell me about your working relationship with [Contracted Partner 1] as it relates to the BHST project? *(Inner Setting – Networks & Communications)*
   1. PROBE: What do you value most about your relationship with [Contracted Partner 1]?
   2. PROBE: What advice or recommendations do you have to strengthen the collaboration with [Contracted Partner 1]?

OC Navigator:

1. What was your initial vision for the OC Navigator? *(Innovation Characteristics – Innovation Source)*
   1. PROBE: What do you hope this project will achieve?
   2. PROBE: How did this change throughout the project?
2. What barriers/challenges do you foresee in achieving your vision for the OC Navigator? *(Characteristics of Individuals – Knowledge & Beliefs About Innovation)*
3. [Contracted Partner 2] is leading the effort on developing the OC Navigator. How familiar are you with their approach? What are some of the strengths of their approach? What are some of the challenges?
4. How, if at all, has leadership at the County supported the planning and eventual implementation of the OC Navigator? Where, if at all, do you see areas where more support is needed? *(Inner Setting – Readiness for Implementation)*
5. How does the OC Navigator fit with the values of OCHCA? How does it fit with currently established workflows (how clinicians, community members, county staff, etc. navigate behavioral health resources)? *(Inner Setting – Innovation Climate – Compatibility)*
6. Can you tell me about your working relationship with [Contracted Partner 2] as it relates to the BHST project? *(Inner Setting – Networks & Communications)*
   1. PROBE: What do you value most about your relationship with [Contracted Partner 2] PROBE: What advice or recommendations do you have to strengthen the collaboration with [Contracted Partner 2]?

Let’s shift gears here a bit and ask you to think more broadly about the BHST Innovation project....

1. Can you tell me about your working relationship with the MHSOAC as it relates to the BHST project? *(Inner Setting – Networks & Communications)*
   1. PROBE: What do you value most about your relationship with MHSOAC?
   2. PROBE: What advice or recommendations do you have to strengthen the collaboration with MHSOAC?
2. Are there any other important relationships we did not ask about specific to the BHST project?
3. Are there any barriers or challenges that we haven’t covered yet that you would like to tell me about? *(Process – Reflecting & Evaluating)*
4. Are there other thoughts and experiences regarding the BHST Innovation project that you would like to share? *(Process – Reflecting & Evaluating)*
